# Behavioural and physiological response to frustration in autistic youth: associations with irritability

**DOI:** 10.1186/s11689-021-09374-1

**Published:** 2021-07-19

**Authors:** Virginia Carter Leno, Georgia Forth, Susie Chandler, Philippa White, Isabel Yorke, Tony Charman, Andrew Pickles, Emily Simonoff

**Affiliations:** 1 Department of Biostatistics and Health Informatics, Institute of Psychiatry, Psychology & Neuroscience, King’s College London, London, UK; 2 Department of Child and Adolescent Psychiatry, Institute of Psychiatry, Psychology & Neuroscience, King’s College London, London, UK; 3 Department of Psychology, Institute of Psychiatry, Psychology & Neuroscience, King’s College London, London, UK; 4 South London and Maudsley NHS Foundation Trust (SLaM), London, UK; 5 Maudsley Biomedical Research Centre for Mental Health, London, UK

**Keywords:** Irritability, Frustration, Physiology, Autism spectrum disorders, Heart rate

## Abstract

**Background:**

Irritability is a common and impairing occurrence in autistic youth, yet the underlying mechanisms are not well-known. In typically developing populations, differences in frustration response have been suggested as important driver of the behavioural symptoms of irritability. Research exploring the role of frustration response as a risk factor for irritability in autistic populations is limited and often uses parent report or observer ratings; objective measures of frustration response appropriate for use in autistic populations are required to advance the field.

**Methods:**

In the current study, fifty-two autistic adolescents aged 13–17 years from a population-based longitudinal study completed an experimental task designed to induce frustration through exposure to periods of unexpected delay. Behavioural (number of button presses) and physiological (heart rate; HR) metrics were collected during delay periods. Irritability was measured using the parent-rated Affective Reactivity Index (ARI). Analyses used mixed-level models to test whether irritability was associated with different slopes of behavioural and physiological response to experimentally induced frustration during the task. Age and baseline HR (for the physiological data only) were included as covariates.

**Results:**

Analyses showed a marginal association between irritability and the slope of behavioural response (incident rate ratio (IRR) =.98, *p*=.06), and a significant association with the slope of physiological response (*b*=−.10, *p*=.04); higher levels of irritability were associated with a dampened behavioural and physiological response, as indicated by flatter slopes of change over the course of the task. The pattern of results largely remained in sensitivity analyses, although the association with physiological response became non-significant when adjusting for IQ, autism symptom severity, and medication use (*b*=−.10, *p*=.10).

**Conclusions:**

Results suggest that the current experimental task may be a useful objective measure of frustration response for use with autistic populations, and that a non-adaptive response to frustration may be one biological mechanism underpinning irritability in autistic youth. This may represent an important target for future intervention studies.

**Supplementary Information:**

The online version contains supplementary material available at 10.1186/s11689-021-09374-1.

## Introduction

Irritability is a trans-diagnostic concept defined as ‘inter-individual differences in proneness to anger that may reach a pathological extent’ [1]. Pathological irritability can have an extremely negative impact on a young person’s education, home life and well-being; in typically developing populations (we use this term to refer to populations without developmental disorders such as autism spectrum disorder), it is associated with increased likelihood of future depression, anxiety and oppositional defiant disorder, and poorer life outcomes (e.g., lower income level and worse physical health) [1]. Within the most recent diagnostic manual, pathological levels of irritability form a major part of the diagnostic criteria for disruptive mood dysregulation disorder (DMDD [2]); this was previously captured by the syndrome of severe mood dysregulation [3]. It should also be noted that irritability is not specific to DMDD; it is also listed as symptom of depression and post-traumatic stress disorder and is closely related to the ‘touchy and easily annoyed’ symptom of oppositional defiant disorder [2].

Autistic youth (the term autistic will be used throughout in keeping with stakeholder preferences), characterised by impairments in social communication abilities and the presence of restricted and repetitive behaviours and interests and sensory differences [2], are found to have elevated rates of irritability [4–6]. In autistic populations, high levels of irritability often manifest as behavioural problems such as oppositional behaviour, aggression, temper tantrums and severe non-compliance [7, 8]. Currently, the mechanisms underpinning variation in irritability in both typically developing and autistic populations are not well understood. Better aetiological understanding will pave the way for more targeted interventions and, given the negative outcomes associated with irritability, promote positive outcomes in autistic youth.

### Experimental correlates of irritability in typically developing youth

Previous work in typically developing children on the cognitive and neural correlates of irritability has primarily implicated alterations in emotion processing and aberrant response to frustration [9, 10]. Aberrant responses to frustration are often studied using laboratory-based tasks that either delay or block goal attainment, normally rewards. Typically developing children with severe irritability (operationalized as meeting criteria for severe mood dysregulation) rate themselves as being more aroused than children without irritability (but no different from children with bipolar disorder) in response to experimentally induced frustration [11]. Typically developing children with severe irritability also show poorer performance on spatial attention tasks and less neural activation to negative feedback in the parietal, parahippocampal, and thalamic/cingulate/striatal regions, during conditions of experimentally induced frustration [12]. In young children, higher levels of irritability are associated with decreased activation in the anterior cingulate and striatum during conditions of frustration [13]. As the research base of studies of frustration and irritability is still relatively limited, one can also look to research with related behavioural phenotypes with high levels of irritability, for example, disruptive behaviour disorders (characterised by aggression, rule-breaking and non-compliance/oppositionality), although it should be noted that irritability is more characteristic of oppositional defiant disorder as compared to conduct disorder. Children with disruptive behaviour disorders (i.e., with a diagnosis of oppositional defiant or conduct disorder) exhibit a blunted physiological response, as indicated by changes in heart rate (HR) or electrodermal activity, to frustration and stress [14, 15]. Furthermore, in youth with disruptive behaviour disorders, higher stress reactivity predicted decline in aggressive behaviour 1 year later [16]. Similarly, adults from the general population with high levels of trait anger also show decreased neural response to experimentally induced frustration [17]. However, meta-analyses find that overall, children and adolescents with conduct problems are characterised by increased HR reactivity [18]. Heterogeneity in the directionality of results may in part be due to differing proportions of youth with conduct vs. oppositional defiant disorder across different samples, and therefore variable levels of irritability.

### Correlates of irritability in autistic youth

Despite the high prevalence of irritability in autistic populations, limited work exists to address underpinning mechanisms, and understand whether the experimental correlates are comparable to those reported from non-autistic (e.g., typically developing) populations. One study found irritability predicted physiological (cortisol and HR) response to stress in autistic youth [19], in that autistic youth with high levels of irritability had a blunted physiological stress response, although results became non-significant when adjusting for levels of anxiety. However, this work focused on response to stress, rather than frustration. In typically developing adults, fear vs. anger in response to stress is associated with distinct biological profiles [20]; thus, responses to frustration may be a clearer correlate of irritability (as compared to anxiety) in autistic youth. Although irritability was not directly measured, another recent study found no association between observer-rated response to frustration and behavioural problems in autistic children [21]. However, the lack of association may have been due in part to limited variance in behavioural problems, as the sample consisted of individuals who had been hospitalized due to severe psychiatric difficulties. Conversely, others find that increases in peak HR moderately predict subsequent episodes of challenging behaviour in autistic children aged 2–4 years [22]. In older autistic children (aged 4–7 years), parent ratings of poorer emotion regulation predicted both higher levels of concurrent behavioural problems and a worsening of behaviour problems at follow-up 1 year later [23].

### Aims

The current paper tests the association between objectively measured response to frustration and irritability in a population-based sample of autistic youth. Reviews of studies of emotional responsivity in autistic populations note the over-reliance on parent or self-report measures (unlike in typically developing populations, where experimental measures are better developed) [24], highlighting the need for objective measures of emotional response and regulation suitable for use in autistic youth. We adapt an existing experimental task [25, 26] designed to evoke frustration, which captures both behavioural and physiological responses, to comprehensively capture individual variation in frustration response. Based on the existing literature, we hypothesised that irritability would be associated with a greater behavioural and physiological response to frustration. Thus, participants with higher levels of irritability would require fewer trials to elicit frustration, and such would display a steeper trajectory of behavioural and physiological response over the duration of the task.

## Method

### Sample

Participants were part of the QUEST follow-up study [27], a longitudinal community-based sample recruited at age 4–8 years (wave 1; *N*=277) and followed up at ages 11–15 years (wave 2; *N*=211) and 13–17 years (wave 3; *N*=214), as part of the wider IAMHealth project. The original target population for the study was all children born in a 4-year period, living in two London boroughs, who had a clinical diagnosis of ASD (*N*=447). Two hundred seventy-seven children were recruited into the study upon entry and selectively stratified into an ‘intensively studied’ (hereafter intensive; *n*=101), who completed a more in-depth protocol of assessments, and ‘extensively studied’ group (hereafter extensive; *n*=176), who completed online questionnaires only, and this sampling structure was maintained at subsequent waves of data collection. The current study focuses on wave 3 intensive group only. See Supplementary Figure 1 for a flow chart of sample recruitment. Although all participants had a clinical diagnosis of autism spectrum disorder, the intensive group had their diagnosis confirmed at wave 2 with the Autism Diagnostic Observation Schedule-2 (ADOS-2 [28]), and a subset also with the Autism Diagnostic Interview-Revised (ADI-R [29]). All participating families gave their written informed consent (from young people themselves if ≥16 years in age and were deemed to have capacity, otherwise from parents or caregivers), and the study was approved by Camden and King’s Cross Ethics Sub-Committee (17/LO/2098 for wave 2, 17/LO/0397 for wave 3). Table 1 gives a comparison of key measures between the full wave 3 intensive sample (*n*=77) versus the wave 3 intensive subsample who completed the experimental frustration task (*n*=52).

**Table 1:** Wave 3 full sample and subsample demographic information

Mean (standard deviation, range)	Full intensive sample (*n*=77)	Sample who completed frustration task (*n*=52)	*t*-test of group differences
Age	15.38 (1.16, 13.2–17.8)	15.40 (1.10, 13.2–17.3)	*p*=.61
% male (*n*)	60% (46)	63% (33)	*p*=.61
IQ^a^	69.88 (31.36, 19–129)	84.54 (21.74, 33–129)	*p*<.001
Autism Severity^a^ (ADOS-CSS)	6.72 (2.66, 1–10)	6.31 (2.83, 1–10)	*p*=.32
ARI total	3.74 (3.26, 0–12)	4.02 (3.37, 0–12)	*p*=.56

^a^Measured at wave 2, approximately 2 years previously

*ADOS-CSS* Autism Diagnostic Observation Schedule-2 calibrated severity score, *ARI* Affective Reactivity Index

## Measures

### Psychiatric symptoms

#### Clinical interview

The Child and Adolescent Psychiatric Assessment-parent version (CAPA [30, 31]) is an interviewer-based structured diagnostic interview for use with children aged 9–17 years. This was used to identify symptoms of psychiatric disorders that had been present in the past 3 months. The current study uses the total count of oppositional defiant disorder (ODD) symptoms (aside from the ‘spiteful/vindictive behaviour’ and ‘blames others’ items as these had <5 endorsements across the whole sample).

### Questionnaires

#### Affective Reactivity Index (ARI)

The parent-rated ARI [32] was used to assess participants’ level of irritability and includes six items relating to feelings/behaviours specific for irritability and one question assessing impairment due to irritability, with a higher score indicative of a higher level of irritability. The internal consistency was examined in the full QUEST sample (wave 2 intensive + extensive; *n*=201) and found to be excellent (*α* = 0.90), and comparable to that reported previously in samples of autistic young people (*α* = 0.82) [19].

#### Aberrant Behavior Checklist (ABC)—irritability subscale

The parent-rated ABC [33] is a measure developed to assess behaviour problems in children with developmental and intellectual disabilities. The 15-item irritability subscale used currently is often used as an outcome measure in clinical trials [32]. The internal consistency of the subscale was examined in the current intensive sample and found to be excellent (*α* = 0.93).

### Direct assessments

#### Baseline HR

Prior to beginning the task battery, participants watched a relaxing video for 5 min to obtain an estimate of their baseline HR. Participants were given a choice of four relaxing nature-themed videos (‘birds’, ‘bunnies’, ‘kaleidoscope’ or ‘northern lights’). Average HR was calculated across the four consecutive 30-s segments of data collected during this period, the first 60 s and the last 120 s were excluded to ensure data quality.

#### Frustration task

**Fig. 1:**
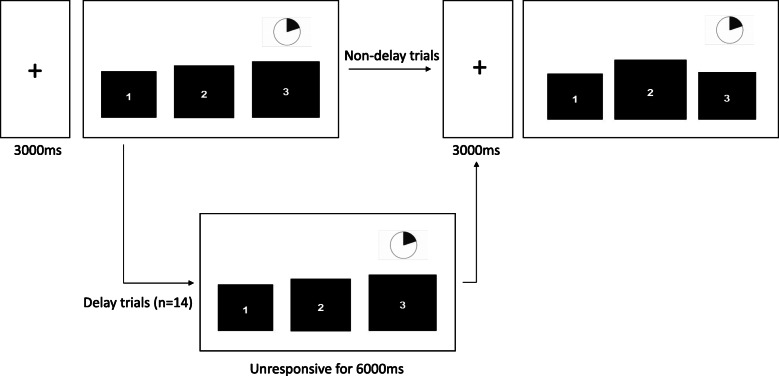
Schematic of frustration task

A novel task was designed and programmed in E-Prime 2.0, based on a previously described delay frustration task [25, 26]. The task was simplified to allow maximum participation in our sample. Participants were asked to select the smallest square from a choice of three. Once participants responded, the task moved directly onto the next trial without any feedback. To motivate participation, participants were informed that most people their age completed around 60 trials, and a pie was shown for each trial to indicate how much time they had left (see Fig. 1 for a schematic of the task). However, during the task, participants experienced several unexpected delays (14 delay trials out of a total of 50 trials), where the computer became unresponsive to their button presses for 6 s. These were pseudo-randomly presented, in that the first six trials were always non-delay trials, and the order of presentation was the same for each participant. The average accuracy of response overall and the number of button presses during each 6-s delay trial were extracted. Overall accuracy was marked as missing for one participant as, from inspection of their pattern of responses, it was clear they had thought the goal was to select the biggest square (as opposed to the smallest). The task lasted approximately 5 min and was part of a wider task battery.

### Physiological data extraction and processing

Electrocardiogram (ECG) data were recorded at 2000Hz using BIOPAC systems MP160 with BioNomadix wireless transmitters. Data was collected and processed using AcqKnowledge 5.0.1 [34]. ECG measurements were collected using electrodes placed in the lead-II position on the back. The ECG signal was filtered using a Comb Band 50-Hz filter to remove electrical noise and a 1-Hz high-pass filter to remove baseline drift and movement artefact. R wave peaks, each representing a heartbeat, were automatically identified and labelled using the AcqKnowledge find cycle protocol. The signal was visually inspected to ensure that R wave peaks had been correctly identified and any movement artefact removed. HR was extracted for each inter-beat interval during the 6-s delay periods. Digital markers indicating the beginning and end of each delay trial were sent via E-Prime, and these were used to demarcate the segments of data extraction.

For both the baseline and experimental task recording, four participants from the 52 who completed the direct assessments had no usable ECG data due to electrode refusal (*n*=2) and corrupted data files (*n*=2). For the baseline recording, ECG segments with more than three consecutive missing peaks or 10% of data missing were excluded (as in previous studies of autistic populations) [35], and participants with ≥ 50% missing task data overall were excluded (*n*=4), leaving a final sample of *n*=44 for the baseline HR data. For the experimental task, ECG task segments with more than one peak missing were excluded, and as before, participants with ≥ 50% missing task data were excluded (*n*=1), leaving a final sample of *n*=47 for the frustration task HR data.

### Statistical analysis

All analyses were conducted in Stata 16. First, bivariate correlations were run between the ARI and demographic characteristics (age, sex, IQ and autism severity), and other measures of irritability (parent-rated ABC irritability subscale and the number of ODD symptoms from the CAPA), to confirm the convergent validity of the irritability construct as measured by the ARI. Next, multilevel mixed-effect models were used to test associations between ARI and trajectories of behavioural and physiological responses during the frustration task. The key term of interest was the time-by-irritability interaction, but we also tested for main effects of irritability, equating to an association with the overall number of presses/HR (rather than the slope of change). Any significant interactions were depicted graphically using predicted marginal means of behavioural and physiological response in low vs. high irritability groups, defined using −1 or +1 standard deviation (SD) from the sample mean; however, all statistical analyses used the continuous form of the ARI. Age was included as a covariate in all task analyses, along with baseline physiology in HR analyses. As the behavioural data was the count of presses during each delay trial, a negative binomial model was specified. Likelihood ratio (LR) tests suggested a model with random intercept, and slope was adequate for HR data, but the addition of a quadratic term of time (time^2^) was necessary for the press data (LR χ^2^(1) = 18.21, *p*<.01). After primary analyses, two sensitivity analyses were conducted. The first added overall task accuracy (averaged across all 50 trials) as a covariate as a proxy for task engagement, and the second included IQ, autism severity and medication status (coded as a binary variable of currently taking medication yes/no; split 45/55% (*n*=23/28); made up of 13% (*n*=3) minor tranquilizers/sedatives, 22% (*n*=5) stimulants, 4% (*n*=1) non-stimulants (e.g., atomoxetine, guanfacine, clonidine), 17% (*n*=4) anti-depressant, 9% (*n*=2) anti-convulsant, 22% (*n*=5) asthma medication, 52% (*n*=12) other medication) to assess the evidence for potential confounders (especially those which may index difficulties in understanding the task) on motor and physiological response. We report unstandardized coefficients throughout (*b*) unless specified.

## Results

Table 2 presents the bivariate correlations between ARI and demographic characteristics and other measures of irritability. ARI was not significantly correlated with age, sex, IQ or autism severity (all *p*>.18). As expected, ARI was significantly correlated with the ABC irritability subscale (*r*=.78, *p*<.001) and the number of ODD symptoms on the CAPA (*r*=.71, *p*<.001).

**Table 2:** Bivariate correlations between demographic characteristics and measures of irritability

	1	2	3	4	5	6	7
ARI total (1)	-						
Age (2)	−.11	-					
Sex (3)	−.06	.04	-				
IQ (4)	.17	−.07	−.03	-			
Autism severity (5) (ADOS-CSS)	.05	−.13	−.14	−.30*	-		
ABC irritability (6)	.78**	−.08	.14	−.15	.18	-	
CAPA number of ODD symptoms (7)	.71**	−.01	−.06	.19	.14	.62**	-

*p<.01, **p<.001

*ADOS-CSS* Autism Diagnostic Observation Schedule-2 calibrated severity score, *ABC* Aberrant Behavior Checklist, *ARI* Affective Reactivity Index, *CAPA* Child and Adolescent Psychiatric Assessment, *ODD* oppositional defiant disorder

### Associations between irritability and response to frustration

### Behavioural response

**Fig. 2:**
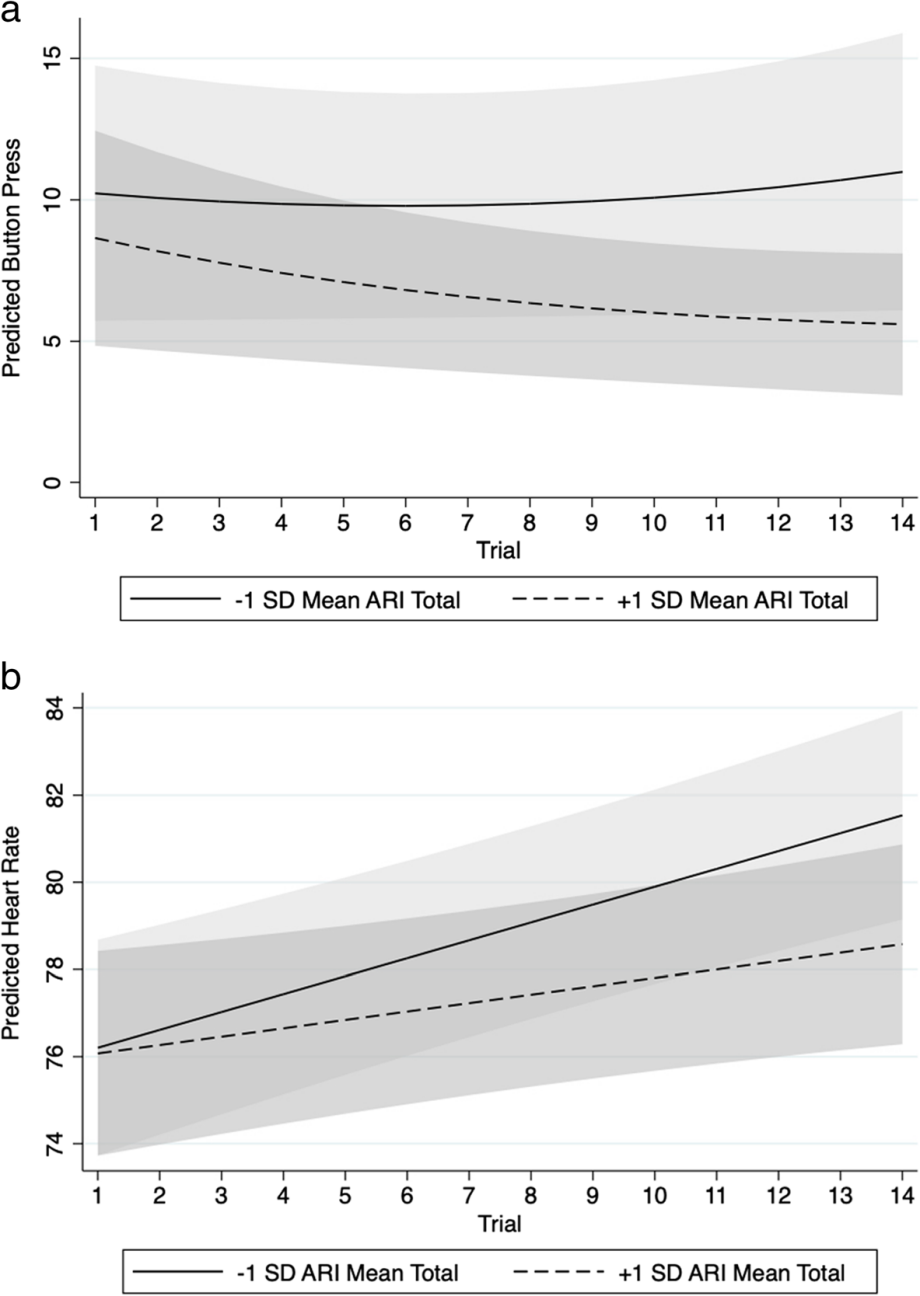
Marginal predicted means of **a** behavioural and **b** physiological response to frustration over the course of the task, split by −/+ 1 standard deviation (SD) from mean ARI total score

The main effects of time (incident rate ratio (IRR) =.95, *p*=.14), time^2^ (IRR=1.01, *p*=.34), age (IRR=.94, *p*=.64) and ARI (IRR=.94, *p*=.13) were all non-significant predictors of number of presses. The time-by-ARI interaction was a marginal but non-significant predictor (IRR=.98, *p*=.06). The time-by-ARI interaction term indicates for a one-point increase in irritability, one would expect increase in the rate of button presses by a factor of 0.98 (i.e., a decrease). This is illustrated in Fig. 2a, where it appears that the high irritability group showed less change in response over the course of the task. Sensitivity analyses found the result did not change when adjusting for overall accuracy (IRR=.98, *p*=.06), and accuracy did not predict responses (IRR=.72, *p*=.62); however, when IQ, autism severity and medication use were included as covariates, the time-by-ARI interaction term was significant (IRR=.98, *p*=.02). Neither IQ (IRR=1.01, *p*=.81) nor autism severity (IRR=1.09, *p*=.07) was significant predictors. Medication use was significantly associated with number of presses (IRR=.45, *p*<.01).

### Physiological response

There were significant main effects of both baseline HR (*b*=.73, *p*<.01) and time (*b*=.95, *p*<.01) in predicting HR, but not ARI (*b*=−.24, *p*=.28) or age (*b*=−.22, *p*=.77). The time-by-ARI interaction (*b*=−.10, *p*=.04) was a significant predictor of HR (Fig. 2b). As with the behavioural data, participants with higher levels of irritability appear to show a flatter slope of HR change over the task. Sensitivity analyses found the result did not change when adjusting for overall accuracy (*b*=−.10, *p*=.05) and that accuracy predicted HR (*b*=−10.30, *p*=.05). Analyses including IQ, autism symptoms and medication use as covariates led the time-by-irritability interaction term to become non-significant (*b*=−.10, *p*=.10). Neither IQ (*b*=−.03, *p*=.40), autism severity (*b*=−.08, *p*=.79) nor medication use (*b*=3.20, *p*=.06) was significant predictors of HR, although the coefficient for medication use was marginal.

## Discussion

Previous work in typically developing children has implicated an aberrant response to frustration as a driver of irritability [9, 10]. In the current study, we adapted an existing experimental paradigm to investigate whether irritability was associated with response to frustration using multi-modal assessment of behavioural and physiological markers, in a well-characterized sample of autistic youth. Results showed that individuals with higher levels of irritability were characterised by a dampened pattern of behavioural and physiological response to frustration, as indicated by flatter slopes of change during the experimental task. The pattern of results largely remained unchanged in sensitivity analyses (although associations between irritability and change in HR during the task became statistically non-significant when adjusting for IQ, autism symptoms and medication use). Results suggest this paradigm shows promise as an objective measure of frustration response in autistic youth; however, replication in larger samples is necessary.

In the current study, we tested the role of response to mild provocation, by adapting an existing task designed to elicit frustration [25, 26] to be suitable for young people with a range of cognitive ability. The task involved exposing participants to a short period of delay, and this delay meant they thought would obtain a lower number of points than most people their age, thus can be situated with the research domain criteria construct of ‘frustrative non-reward’ [36, 37]. The current task is also relevant to the field of emotion regulation, where recent reviews have noted the overreliance on parent or self-report measures of response to stressors in autistic populations, which may lead to results which are in part due to shared method variance [24]. We note here that the task was completed by autistic youth with a wide range of functioning (IQ range of 33–129), and adjusting for accuracy and IQ did not change the pattern of behavioural results, suggesting this experimental paradigm is likely applicable to more representative autistic samples than are typically included in experimental research. Sensitivity analyses adjusting for IQ, autism symptoms and medication use did cause associations between physiological metrics of frustration and irritability to become statistically non-significant (although the beta coefficient was unchanged, suggesting the change in *p* value may have been in part due to an increase in standard error with the inclusion of additional covariates and lower sample size); future work is required to replicate the current findings in larger and therefore better powered samples.

Previous work has used experimental paradigms to test associations between irritability and response to stress in autistic youth [19, 38]; however, frustration and stress elicit different biological responses [20], and thus the relevance of each domain to irritability in autistic youth should be studied independently. We had hypothesised that individuals with irritability would show a greater response to frustration; however, from inspection of plots of button presses over the course of the task (Fig. 2a), it appears that participants with higher levels of irritability did not show an increase in the number of button presses over time to delay trials (unlike those with lower levels of irritability). This is contrary to our predictions, as previous research reports young adults with higher levels of attention-deficit hyperactivity disorder (ADHD) traits press the button more often on the delay trials [39] and children with ADHD find delay tasks more aversive [40], and a substantial proportion of young people with ADHD also have high levels of irritability [41]. Therefore, we hypothesised that participants with irritability would find the delay more frustrating and press the button more when the computer became unresponsive. One interpretation is that a less steep slope of behavioural response reflects disengagement in the face of early frustration; participants with higher levels of irritability may have been less willing to continue to try and move the trials on when they appeared to get stuck. The shallower slope of behavioural response could reflect a sadness-type withdrawal due to dysphoria (which often co-occurs with reactivity in youth with emotional dysregulation [42]), prompted by the idea of doing badly compared to peers. Differences in task presentation may also be important to consider; compared to the original version of the task, we used simplified stimuli (selecting the smallest square from a choice of three) to allow for maximal participation. This may have led to a less engaging task for participants with higher IQ, so leading to less intense feelings of frustration, although this cannot explain the association with irritability. Further work combining objective behavioural measures such as button presses with observational coding of behaviour and subjective reports of mood is needed to clarify the meaning of behavioural metrics in this task (although we highlight the potential limitations of relying on self-rated mood states in populations of individuals who may have difficulty differentiating and labelling high arousal states). Additionally, collecting information on how irritability relates to responses during other high arousal states (e.g., anxiety) would also be informative in terms of pinpointing which types of emotional states contribute to behavioural irritability.

In addition to associations with behavioural response, irritability was also associated with a different pattern of physiological response (as measured by change in HR) to frustration over the course of the task. Contrary to our predictions, results suggested that higher levels of irritability were associated with a flatter HR slope across the task. This blunted response is similar to findings from populations of typically developing youth characterised by high levels of irritability, mainly youth with disruptive behaviour disorders (e.g., ODD, conduct disorder), who exhibit a blunted physiological response to frustration [14, 15]. Similarly, general population studies find high levels of trait aggression are associated with decreased neural response to experimentally induced frustration, especially in the frontal and limbic regions [17]. Interestingly, previous work has also found irritability is associated with a blunted cortisol and HR response to stress in autistic samples of a similar age [19], and this effect was largely accounted for by co-occurring anxiety. In typically developing populations, a blunted response to frustration has been interpreted as indicative of an under-active fear system, which could lead to impairments in fear-conditioning, stimulation seeking and risk-taking behaviours. We highlight that comparisons should be made with caution as the studies cited above did not measure irritability, but are useful to consider because they have studied groups with symptoms that are very similar to the behavioural manifestation of irritability in autistic populations (e.g., oppositionality, aggression, severe non-compliance). Subtyping the types of behavioural difficulties under study may be key to understanding mixed findings: higher arousal is related to reactive aggression and anxiety, whereas lower arousal is more often associated with proactive aggression in typically developing youth [43]. Further work is needed to better characterise the nature of irritability and its behavioural manifestations in autistic vs. typically developing populations, in order to facilitate comparisons of aetiological mechanisms between the two groups.

A different interpretation is that the blunted physiological response in those with high irritability may have been due to ceiling effects, in that those participants with high levels of irritability were already feeling frustrated due to completing a battery of other experimental tasks, and thus they had ‘used up’ any event-specific physiological responsiveness by the time they completed a task deliberately designed to elicit frustration. In the current project, a decision was made to administer the frustration-eliciting task at the end of the task protocol in case it elicited any severely negative responses, but this may have inadvertently diluted the magnitude of frustration-specific responses. Future work should consider completing a baseline measurement of physiological arousal directly before administering tasks designed to elicit specific affective states to better understand whether changes in physiology can be directly attributed to a given experimental task.

### Strengths and limitations

To our knowledge this is one of the few studies to collect objective measurements of frustrative response in autistic youth. We explicitly designed the experimental task and selected the parent-rated measures to answer the question of whether irritability is associated with aberrant frustration response in autistic youth; the current paper presents a succinct test of this a priori hypothesis. Although the task has not been used in autistic populations before (but is often used in ADHD populations), the significant increase in HR over the course of the task and associations between both button press and HR and parent-rated irritability suggest the task was evoking frustration as designed to. Furthermore, the task was completed by a sample with a wide range of IQ, with minimal data loss, and the pattern of results were largely unchanged when adjusting for overall accuracy (which could be considered a proxy for task engagement), IQ, severity of autism symptoms and medication use (although associations between irritability and HR became statistically non-significant), suggesting the task may be appropriate for heterogeneous samples of autistic youth. However, it could be that the source of frustration depends in part on intellectual level; participants with higher IQ may have found the task frustrating because they pictured themselves failing against an imaginary peer, whereas those with lower IQ may have struggled to hold this representation of ‘the average score’ in mind, and simply found the task frustrating because of the delay. Future work that systematically varies the social comparison aspect of this task (e.g., for some sections of the task participants are told their scores will be compared against a peer, whereas other sections they are informed they are simply testing their ability, but still experience delays) may shed light on whether the drivers of frustration differ between autistic youth with and without intellectual disability, and how integral the social comparison aspect is to generating frustration. Given that reviews of the field have noted that research on emotion regulation and reactivity in autistic youth relies on questionnaire data [24], where parent-report measures may not fully capture emotional response, and self-report measures are not appropriate for very young children, or individuals who are minimally verbal and/or have significant levels of alexithymia, the development of valid experimental measures is an important goal.

We also highlight our statistical approach; modelling change over time in the frustration task indicated that autistic individuals with irritability are characterised by a different trajectory of behavioural and physiological response to frustration. Averaging across all trials would have likely missed these differences that may be key to understanding the neurobiological basis of irritability. Additionally, the current sample was community-based (as opposed to samples recruited through current clinical attendance, which are known to be enriched for symptom severity), thus making it more representative of autism as a whole, and all participants had their diagnosis confirmed with ‘gold-standard’ diagnostic instruments. Although the subsample that completed the experimental task had a higher IQ than the full community-based sample, in all other key variables there were no significant differences. In terms of limitations, the lack of control group means whether a similar pattern of associations between cognition and behaviour are found in typically developing samples cannot be tested. We also acknowledge use of a moderately sized sample, which could have led to limited power to detect associations of smaller effect.

## Conclusions

Current results suggest that a maladaptive response to frustration may be one mechanism that underpins irritability and its behavioural manifestations (e.g., aggression, temper tantrums, challenging behaviours) in autistic populations. Further clarification, using multi-modal methodologies (e.g., observational and direct measurements paired with questionnaires), is required as to better understand whether irritability in autistic youth is characterised by hypo- or hyper-responsiveness to frustrating situations. This in turn will guide targets for future interventions, for example, cognitive reappraisal and relaxation techniques [44]. Given the poor outcomes for youth with severe irritability [1], it is imperative to understand the mechanisms by which autistic individuals develop irritability, and therefore how best to intervene. This paper presents an objective measure of frustration response and emotion regulation for use with autistic populations, and therefore is an important step towards this mechanistic understanding.

## Supplementary Information

**Additional file 1: Supplementary Figure 1.** Overview of QUEST Follow-Up Study

## Data Availability

The datasets used and/or analysed during the current study are available on reasonable request.

## Abbreviations

ABC: Aberrant behavior checklist
ADHD: Attention deficit hyperactivity disorder
ADI-R: Autism diagnostic interview–revised
ADOS-CSS: Autism diagnostic observation schedule-2 calibrated severity score
ARI: Affective reactivity index
CAPA: Child and adolescent psychiatric assessment
DMDD: Disruptive mood dysregulation disorder
ECG: Electrocardiogram
HR: Heart rate
LR: Likelihood ratio
IQ: Intellectual quotient
IRR: Incident rate ratio
ODD: Oppositional defiant disorder

## References

[1] Vidal-Ribas P, Brotman MA, Valdivieso I, Leibenluft E, Stringaris A (2016). The status of irritability in psychiatry: a conceptual and quantitative review. J Am Acad Child Adolesc Psychiatry..

[2] American Psychological Association (2013). Diagnostic and statistical manual of mental disorders.

[3] Leibenluft E (2011). Severe mood dysregulation, irritability, and the diagnostic boundaries of bipolar disorder in youths. Am J Psychiatry..

[4] Mayes SD, Calhoun SL, Murray MJ, Ahuja M, Smith LA (2011). Anxiety, depression, and irritability in children with autism relative to other neuropsychiatric disorders and typical development. Rese Autism Spectr Disord..

[5] Simonoff E, Jones CRG, Pickles A, Happé F, Baird G, Charman T (2012). Severe mood problems in adolescents with autism spectrum disorder. J Child Psychol Psychiatry..

[6] Mandy W, Roughan L, Skuse D (2014). Three dimensions of oppositionality in autism spectrum disorder. J Abnorm Child Psychol..

[7] Gadow KD, Devincent CJ, Pomeroy J, Azizian A (2005). Comparison of DSM-IV symptoms in elementary school-age children with PDD versus clinic and community samples. Autism..

[8] Maskey M, Warnell F, Parr JR, Le Couteur A, McConachie H (2013). Emotional and behavioural problems in children with autism spectrum disorder. J Autism Dev Disord..

[9] Leibenluft E (2017). Pediatric irritability: a systems neuroscience approach. Trends Cogn Sci..

[10] Leibenluft E, Stoddard J (2013). The developmental psychopathology of irritability. Dev Psychopathol..

[11] Rich BA, Mariana S, Perez-Edgar KE, Fox N, Pine D, Leibenluft E (2007). Different psychophysiological and behavioral responses elicited by frustration in pediatric bipolar disorder and severe mood dysregulation. Am J Psychiatry..

[12] Deveney CM, Connolly M, Haring CT, Bones BL, Reynolds RC, Kim P (2013). Neural mechanisms of frustration in chronically irritable children. Am J Psychiatry..

[13] Perlman SB, Jones BM, Wakschlag LS, Axelson D, Birmaher B, Phillips ML (2015). Neural substrates of child irritability in typically developing and psychiatric populations. Dev Cogn Neurosci..

[14] Fairchild G, van Goozen SHM, Stollery SJ, Brown J, Gardiner J, Herbert J, Goodyer IM (2008). Cortisol diurnal rhythm and stress reactivity in male adolescents with early-onset or adolescence-onset conduct disorder. Biol Psychiatry..

[15] Northover C, Thapar A, Langley K, Fairchild G, van Goozen SHM (2016). Cortisol levels at baseline and under stress in adolescent males with attention-deficit hyperactivity disorder, with or without comorbid conduct disorder. Psychiatry Res..

[16] Schoorl J, van Rijn S, de Wied M, van Goozen SHM, Swaab H (2017). Neurobiological stress responses predict aggression in boys with oppositional defiant disorder/conduct disorder: a 1-year follow-up intervention study. Eur Child Adolesc Psychiatry..

[17] Pawliczek CM, Derntl B, Kellermann T, Gur RC, Schneider F, Habel U (2013). Anger under control: neural correlates of frustration as a function of trait aggression. Plos One..

[18] Lorber MF (2004). Psychophysiology of aggression, psychopathy, and conduct problems: a meta-analysis. Psycholog Bull..

[19] Mikita N, Hollocks MJ, Papadopoulos AS, Aslani A, Harrison S, Leibenluft E, Simonoff E, Stringaris A (2015). Irritability in boys with autism spectrum disorders: an investigation of physiological reactivity. J Child Psychol Psychiatry..

[20] Moons WG, Eisenberger NI, Taylor SE (2010). Anger and fear responses to stress have different biological profiles. Brain, Behavior, and Immunity..

[21] Northrup JB, Goodwin M, Montrenes J, et al. Observed emotional reactivity in response to frustration tasks in psychiatrically hospitalized youth withautism spectrum disorder. Autism. 2020;24(4):968-982. 10.1177/1362361320908108 PMC7700748 32169018

[22] Nuske HJ, Finkel E, Hedley D, Parma V, Tomczuk L, Pellecchia M, Herrington J, Marcus SC, Mandell DS, Dissanayake C (2019). Heart rate increase predicts challenging behavior episodes in preschoolers with autism. Stress..

[23] Berkovits L, Eisenhower A, Blacher J (2017). Emotion regulation in young children with autism spectrum disorders. J Autism Dev Disord..

[24] Cai RY, Richdale AL, Uljarević M, Dissanayake C, Samson AC (2018). Emotion regulation in autism spectrum disorder: where we are and where we need to go. Autism Res..

[25] Bitsakou P, Antrop I, Wiersema JR, Sonuga-Barke EJS (2006). Probing the limits of delay intolerance: preliminary young adult data from the Delay Frustration Task (DeFT). J Neurosc Methods..

[26] Wilbertz G, Trueg A, Sonuga-Barke EJS, Blechert J, Philipsen A, Tebartz van Elst L (2013). Neural and psychophysiological markers of delay aversion in attention-deficit hyperactivity disorder. J Abnorm Psychol..

[27] Salazar F, Baird G, Chandler S, Tseng E, O’sullivan T, Howlin P, Pickles A, Simonoff E (2015). Co-occurring psychiatric disorders in preschool and elementary school-aged children with autism spectrum disorder. Journal of autism and developmental disorders..

[28] Lord C, Rutter M, DiLavore P, Risi S, Gotham K, Bishop S (2012). Autism diagnostic observation schedule second edition (ADOS-2).

[29] Rutter M, Le Couteur A, Lord C (2003). The Autism Diagnostic Interview-Revised.

[30] Angold A, Costello EJ (2000). The Child and Adolescent Psychiatric Assessment (CAPA). J Am Acad Child Adolesc Psychiatry..

[31] Angold A, Prendergast M, Cox A, Harrington R, Simonoff E, Rutter M (1995). The Child and Adolescent Psychiatric Assessment (CAPA). Psychol Med..

[32] Stringaris A, Goodman R, Ferdinando S, Razdan V, Muhrer E, Leibenluft E, Brotman MA (2012). The Affective Reactivity Index: a concise irritability scale for clinical and research settings. J Child Psychol Psychiatry..

[33] Aman M, Singh N (1994). The Aberrant Behavior Checklist-Community.

[34] BIOPAC Systems I. (2016). AcqKnowledge 5.0.1.

[35] Bazelmans T, Jones EJH, Ghods S, Corrigan S, Toth K, Charman T, Webb SJ (2019). Heart rate mean and variability as a biomarker for phenotypic variation in preschoolers with autism spectrum disorder. Autism Research..

[36] Insel T, Cuthbert B, Garvey M, Heinssen R, Pine DS, Quinn K, Sanislow C, Wang P (2010). Research domain criteria (RDoC): toward a new classification framework for research on mental disorders. Am J Psychiatry..

[37] National Institute of Mental Health. Research Domain Criteria Matrix. 2020. https://www.nimh.nih.gov/research/research-funded-by-nimh/rdoc/constructs/rdoc-matrix.shtml. Accessed 2^nd^ October 2020.

[38] Hollocks MJ, Pickles A, Howlin P, Simonoff E (2016). Dual cognitive and biological correlates of anxiety in autism spectrum disorders. J Autism Dev Disord..

[39] Aman MG, Novotny S, Samango-Sprouse C, Lecavalier L, Leonard E, Gadow KD, King BH, Pearson DA, Gernsbacher MA, Chez M (2004). Outcome measures for clinical drug trials in autism. CNS Spectr..

[40] Sonuga-Barke E, Bitsakou P, Thompson M (2010). Beyond the dual pathway model: evidence for the dissociation of timing, inhibitory, and delay-related impairments in attention-deficit/hyperactivity disorder. J Am Acad Child Adolesc Psychiatry..

[41] Stringaris A, Goodman R (2009). Three dimensions of oppositionality in youth. J Child Psychol Psychiatry..

[42] Mazefsky C, Yu L, Pilkonis P. Psychometric properties of the emotion dysregulation inventory in a nationally representative sample of youth. J Clin Child Adolesc Psychology. 2020:1–13. 10.1080/15374416.2019.1703710. 10.1080/15374416.2019.1703710 PMC7781089 31910035

[43] Schoorl J, Van Rijn S, De Wied M, Van Goozen SHM, Swaab H (2016). Variability in emotional/behavioral problems in boys with oppositional defiant disorder or conduct disorder: the role of arousal. Eur Child Adolesc Psychiatry..

[44] Lochman J, Barry T, Pardini D, Kazdin AE, Weisz JR (2003). Anger control training for aggressive youth. Evidence-based psychotherapies for children and adolescents.

