# Supplementary material for: Behavioural and physiological response to frustration in autistic youth: associations with irritability
Source: J Neurodev Disord. 2021 Jul 19;13:27. doi: 10.1186/s11689-021-09374-1 (PMC8287810; doi:10.1186/s11689-021-09374-1)
Supplement: Supplementary file 1 — Additional file 1: Supplementary Figure 1. Overview of QUEST Follow-Up Study [file 11689_2021_9374_MOESM1_ESM.docx]

**Supplementary Figure 1. Overview of QUEST Follow-Up Study**

**W3 Intensive sample participation**

N = 77 (76% of Wave 1 ppts)

60% male (46 male, 31 female)

*Seen by November 2018*

**W3 Extensive sample participation**

N = 137 (78% of Wave 1 ppts)

96% male (132 male, 5 female)

*Completed by November 2018*

**Eligible for Wave 2**

N=277 (all Wave 1 ppts)

**W2 Extensive sample participation**

N = 128 (73% of Wave 1 ppts)

96% male (123 male, 5 female)

*Completed by November 2016*

**W2 Intensive sample participation**

N = 83 (82% of Wave 1 ppts)

57% male (47 male, 36 female)

*Seen by November 2016*

**Eligible for Wave 3**

N = 253

(all Wave 1 participants, excluding Wave 2 decliners (n=10) and Wave 2 no contact (14))

**Validating Autism Dx with ADI-R**

66 cases met criteria for “ADI required”

50 (76%) ADIs completed

->No cases excluded (all met ADI or ADOS criteria)

**W1 Intensive Sample**

N=101

56% male (57 male, 44 female)

*Seen by September 2010*

**W1 Extensive Sample**

N 176

97% male (170 male, 6 female)

*Completed by September 2010*

**Target population**

Children with an ASD diagnosis, born 01/09/2000- 01/09/2004, living in Bromley or Lewisham

N=447

**Participated in Wave 1**

N = 277 (62.0% of target population)

82.0% male

**Selected for Wave 1 Intensive assessment (N =131)**

All girls (n=50)

Random sample of boys (n=81), stratified on: (i) IQ(</>70), (ii) borough, (iii) age (<6.7/>6.8yrs), (iv) SCQ(</>22)

n=131

**Validating Autism dx**

10 cases with very low SCQ (score<10)

Clinical records checked with local clinicians, all dx confirmed -> none excluded.

**W3 completed frustration task**

N = 52

63% male (33 male, 19 female)
